# Novel Observation: Northern Cardinal (*Cardinalis cardinalis*) Perches on an Invasive Jorō Spider (*Trichonephila clavata*) Web and Steals Food

**DOI:** 10.3390/insects13111049

**Published:** 2022-11-13

**Authors:** Arty Schronce, Andrew K. Davis

**Affiliations:** 1 Independent Researcher, 263 Berean Avenue, Atlanta, GA 30316, USA; 2 Odum School of Ecology, University of Georgia, Athens, GA 30602, USA

**Keywords:** *Trichonephila clavata*, jorō spider, invasive, northern cardinal, web strength

## Abstract

**Simple Summary:**

A spider native to east Asia (jorō spider) is spreading in the United States, and a pressing question is how it will affect native fauna. This brief report details an observation of a bird that foraged for food from a jorō web, all while perching on it. This demonstrates one small (positive) impact of the spider, and also emphasizes just how strong its webs are. Additional experimental data on web strength confirms that the webs are capable of supporting a similarly-sized songbird.

**Abstract:**

An invasive spider (*Trichonephila clavata* [L. Koch 1878], or jorō spider) is rapidly expanding throughout the southeast of the United States, engendering many questions about how native fauna will be affected. Here, we describe an observation of a northern cardinal (*Cardinalis cardinalis*, L.) consuming prey items from a jorō web, which serves as an example of a native species deriving a (small) benefit from this new invader. Moreover, the manner of the kleptoparasitism is also noteworthy; the cardinal perched directly on the web, which supported its weight (which is 42–48 g in this species). This appears to be the first documented case of a spider web supporting a perching bird. We also include measurements of other jorō webs, where web strength had been assessed using a force gauge, which revealed that typical webs can support masses up to 70 g before collapsing. Collectively, this information adds to the small but growing body of knowledge about the biology of this non-native spider.

## 1. Background

Spiders make webs to procure food for themselves, although these same webs, and their trapped prey, are sometimes exploited by other species. For example, species of *Argyrodes* spiders reside on or near webs of other orb-weavers and consume trapped insects (a behavior known as kleptoparasitism) [1,2]. Certain species of predatory fireflies have been observed stealing trapped fireflies from spider webs, to sequester their chemical defenses [3]. There are species of hover wasps that consume trapped prey in orb webs [4]. Further, avian kleptoparasitism of insects from spider webs has also been documented; Waide and Hailman [5] described multiple reports of birds that were observed hovering next to spider webs while gleaning the trapped insects (or insect carcasses). These reports included a bunting, a vireo, a warbler, a wren and a hummingbird. Parrish [6] also described instances of hummingbirds stealing food from spider webs in Utah. In addition to this published literature, there are a variety of anecdotal observations and videos on the internet of various songbirds stealing prey items from spider webs. We note that throughout all of these published and anecdotal cases, the birds in question were observed hovering near the webs or perched on branches nearby. The following report describes an observation of a bird native to North America procuring food from a non-native spider web in a very unusual way, by perching directly on the web.

**Figure 1:**
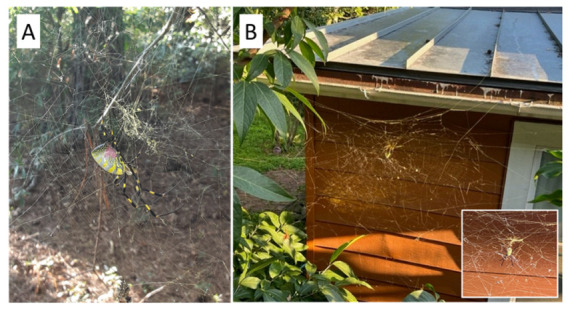
(**A**) A female jorō spider, *Trichnephila clavata*, in its web. Note the complex (not two-dimensional) web pattern. Photo taken by A. Davis in Watkinsville, GA on 18 October 2020. (**B**) Photograph of the jorō spider web in Atlanta where the observation occurred. The web was strung between stalks of wintersweet and a neighboring structure and was approximately 1.25 m × 1.25 m in size.

Jorō spiders, *Trichnophila clavata* L. Koch 1878 (Figure 1A), are an orb-weaving species native to Japan and eastern Asia but have recently been introduced to the southeast United States, being fist observed in 2013 in a few locations in northern Georgia [7] and are now expanding their range. They are expected to continue spreading beyond the southeast, since their physiology appears suited for surviving the colder climates of the north of the United States [8]. This spider species is large (with outstretched legs, up to 10 cm), and they have a striking color pattern of black, yellow and red (Figure 1A). Importantly, their webs are very conspicuous; they are typically up to 1 m in diameter, with a three-dimensional structure (Figure 1B). In the new United States region, these webs are often found in human-dominated landscapes, such as urban areas, and can be built on a wide range of human structures or close vegetation (Davis, pers. obs.). Additionally, of note, is that the individual web fibers are exceptionally strong; spiders in this genus are known for producing silk with high tensile strength [9,10,11]. The following observation also speaks to this exceptional fiber strength.

## 2. Observation

The observation in question took place at the residential home of the first author, in Atlanta, GA, which is an area where jorō spiders have successfully expanded into. There, a jorō spider had built a web next to the side of the house facing the neighboring house, and it had used stalks of wintersweet as support on one side, and the neighboring house on the other (Figure 1B). Based on measurements by the author, this web was approximately 1.25 m × 1.25 m in size, and it was approximately 2 m off the ground. From anecdotal observations of other jorō webs, this web size, structure, and placement appears to be typical of the species in its new range (Davis, pers. obs.). Importantly, jorō spider webs are not completely circular, but tend to have a flattened or level section on the top edge, as is visible in Figure 1B. Presumably, these threads provide structural support.

**Figure 2:**
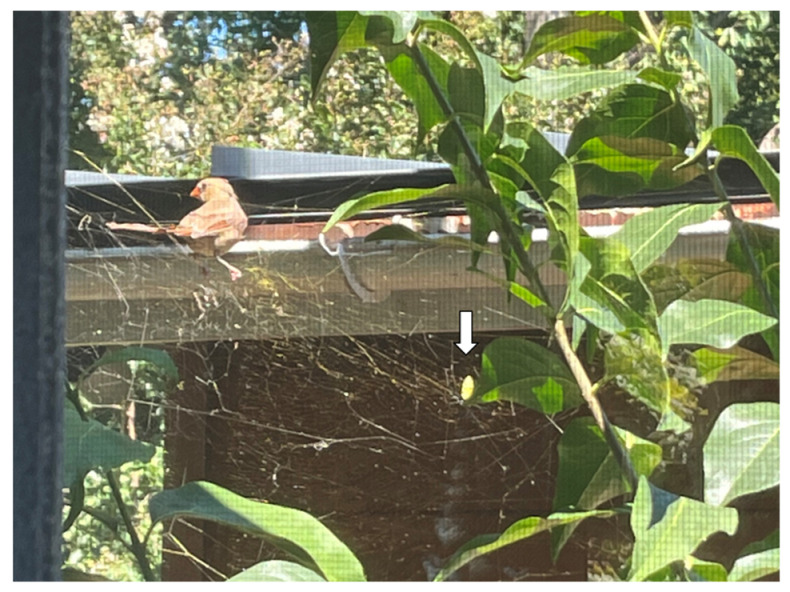
Kleptoparasitism behavior by a northern cardinal (*Cardinalis cardinalis*), observed on 13 September 2022 in Atlanta, GA (Fulton Co.). The cardinal perched on the top of the jorō spider web (which did not break) and proceeded to pick off trapped insects from the web. The spider (arrow) moved to the edge of the web during the encounter. The bird flew off after ~2 min, seemingly without effort or entanglement. The web remained intact and was present the following day. Photo taken by A. Schronce (through a screen door).

On 13 September 2022, the first author observed a female cardinal (*Cardinalis cardinalis*, L.) perched on the top support strands of the web (the web was visible from a screen door). The author did not witness at what point it alighted, but presumably it was not long before the observation. At first, the author believed the bird had been trapped, but this turned out not to be the case. The bird was in fact perched on the web itself (not a branch or support structure), and near the middle of the web, which appeared capable of supporting the bird. The author made sure this was the case by watching closely, and also by examining the web afterward. The author was able to take photos of the perched bird through a screen door (Figure 2). The author observed the cardinal lunge toward the spider (while perched), though the spider moved away from the bird. It is unclear if this was an attempt to capture the spider or to warn it off.

Next, the cardinal proceeded to “glean” from the web, by pecking at (and eating) discarded insect carcasses and/or trapped prey items, all while still perched on the top strands. This behavior lasted for approximately 2 min. Then, the cardinal flew off the web, seemingly without effort or entanglement. After closer inspection, the web itself was not apparently damaged from this event, and the spider was observed in the (undamaged) web the next day. As of the time of this writing (14 October 2022), the spider and web were still present.

## 3. Additional Data in Support of the Observation

**Figure 3:**
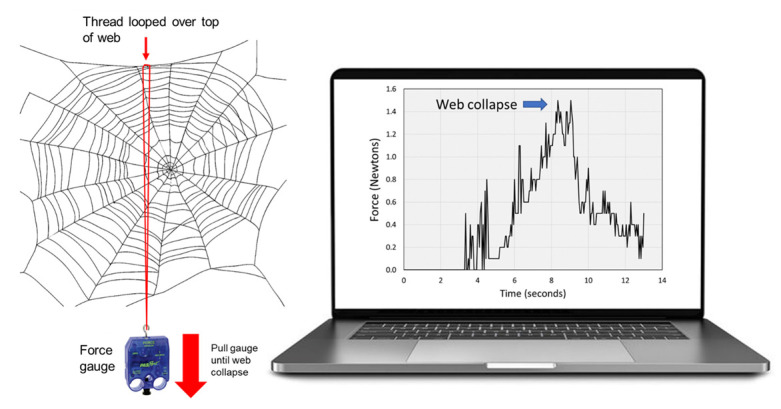
Schematic diagram showing how jorō web strength was measured, on 10 webs in Oconee Co., Georgia. One of the authors (Davis) looped a light thread around the middle of a given web, then tied the lower end of the loop to a handheld, digital force gauge. The gauge was pulled downward until the web collapsed, and the peak force (in Newtons) at which the web broke was recorded on a laptop. Raw force data from one web test is shown for illustration.

To help substantiate the observation above (of a jorō web supporting the weight of a northern cardinal), one of the authors (Davis) drew upon a previously conducted, unpublished set of measurements of actual jorō web strength. In the fall of 2021, the author and an assistant had measured some of the many jorō webs surrounding and/or near his own home in Oconee County, GA, as part of another project. A total of 10 webs of similar size as the Atlanta web had been selected (approximately 1 m × 1 m). For each measurement, a fine thread was looped over a given web (near the midpoint) so that it encircled the web, then the bottom end of the thread loop was attached to an electronic force gauge (Pasco Passport Force Sensor, Pasco.com; Figure 3). The gauge was pulled downward until the web broke; the point at which it broke was recorded, in Newtons, on a laptop computer. Note that this measurement was not an index of individual fiber tensile strength, but rather a metric of the downward force that a bird would apply on the collective web, if perched in the middle (as the cardinal did). From measurements of 10 webs, the average force required to collapse the web was 0.68 N (sd = 0.42, range: 0.2 to 1.5 N), which is equivalent to a downward weight of 69 g.

## 4. Discussion

There are two interesting elements of this observation, which should each add to the small but growing body of knowledge of the biology of the newly invasive jorō spider in the United States. First, the fact that a full-sized northern cardinal (which typically weighs 42–48 g, [12]) could perch on this spider’s web without it breaking appears to be a scientific first; to our knowledge, this represents the first documented case of a spider web (of any species) supporting a perching bird, and it underscores just how strong the webs of this particular species are. In fact, the additional data provided on jorō web strength does confirm that an average web should be capable of supporting a bird of that size.

Spiders in the genus *Trichonephila* have long been studied for the biomechanical properties of their silk, which is exceptionally strong [9,10,11,13,14,15,16]. Throughout this body of literature, we note that the means of measuring silk strength has usually involved testing tensile strength of individual silk fibers using lab-based machinery (e.g., [15,16,17]), which is no doubt necessary for accuracy and repeatability. However, these individual fiber measurements are not easily extrapolated to provide estimates of whole-web strength, since webs of different species (and individuals) can vary in complexity, size, and number of support strands. It is possible to evaluate whole-web mechanical strength using mathematical models [18], which is beyond the scope of this paper, but such a task would be daunting with *T. clavata*, given the complexity of their webs. Moreover, in some species there is even variation in fiber strength and elasticity across the different parts of web, from the fibers that make up the outer frame, to the inner catchment spiral [19]. We further note that the complex, 3-dimensional structure of jorō webs, in particular (with many supporting and anchoring threads), may actually enhance their web strength even further than their fiber strength provides. Note that the web in question (Figure 1B and Figure 2) has a complex design with a variety of support strands.

The other noteworthy aspect of this observation is that it demonstrates at least one way in which a native species could receive a small, but positive benefit from this newly invasive spider; in this one instance, the cardinal apparently utilized the jorō web as a one-time food resource. Cardinals have not previously been reported to eat from spider webs [5]; perhaps in the presence of the sheer numbers of these new webs, along with their large size and abundance of trapped prey, birds, such as cardinals, may learn to exploit this resource. Interestingly, one of the authors (Davis) has also observed native dewdrop spiders (genus *Argyrodes*) in jorō spider webs around his home; these spiders are known kleptoparasites, and even of jorō spiders in their native range [1]. The extent to which the jorō spider webs in North America become hosts to dewdrop spiders deserves further study, as does the use of their webs by native birds.

The jorō spider is rapidly expanding its range in the southeast of the United States, and will likely continue throughout a large portion of the United States, based on its physiology [8]. Therefore, understanding how it will impact the native fauna (positively or negatively) is a priority for research. Such studies are currently underway but are only in their infancy. The information presented in this report should help to further this overarching goal of understanding how this non-native spider will impact the native ecosystem of North America.

## Data Availability

The force measurements presented in this study are available on request from the corresponding author.

## References

[1] Miyashita T. (2001). Competition for a limited space in kleptoparasitic *Argyrodes* spiders revealed by field experiments. Popul. Ecol..

[2] Agnarsson I. (2003). Spider webs as habitat patches—The distribution of kleptoparasites (*Argyrodes, theridiidae*) among host webs (Nephila, tetragnathidae). J. Arachnol..

[3] Faust L., De Cock R., Lewis S. (2012). Thieves in the night: Kleptoparasitism by fireflies in the genus *Photuris* Dejean (Coleoptera: Lampyridae). Coleopt. Bull..

[4] Rossler D.C., Ogan S., Curio E., Krehenwinkel H. (2019). Ability makes a thief: Vision, learning, and swift escape help kleptoparasitic hover wasps not to fall prey to their spider hosts. Behav. Ecol. Sociobiol..

[5] Waide R.B., Hailman J.P. (1977). Birds of five families feeding from spider webs. Wilson Bull..

[6] Parrish J.R. (1988). Kleptoparasitism of insects by a broad-tailed hummingbird. J. Field Ornithol..

[7] Hoebeke E.R., Huffmaster W., Freeman B.J. (2015). *Nephila clavata* L Koch, the Joro Spider of East Asia, newly recorded from North America (Araneae: Nephilidae). PeerJ.

[8] Davis A.K., Frick B.L. (2022). Physiological evaluation of newly invasive joro spiders (*Trichonephila clavata*) in the southeastern USA compared to their naturalized cousin, *Trichonephila clavipes*. Physiol. Entomol..

[9] Higgins L., Rankin M.A. (1999). Nutritional requirements for web synthesis in the tetragnathid spider *Nephila clavipes*. Physiol. Entomol..

[10] Kitagawa M., Kitayama T. (1997). Mechanical properties of dragline and capture thread for the spider *Nephila clavata*. J. Mater. Sci..

[11] Moon M.J. (2018). Fine structure of the aggregate silk nodules in the orb-web spider *Nephila clavata*. Anim. Cells Syst..

[12] Halkin S.L., Shustack D.P., De Vries M.S., Jawor J.M., Linville S.U., Poole A.F., Gill F.B. (2021). Northern Cardinal (*Cardinalis cardinalis*), version 2.0. Birds of the World.

[13] Ebenstein D.M., Wahl K.J. (2006). Anisotropic nanomechanical properties of *Nephila clavipes* dragline silk. J. Mater. Res..

[14] Rousseau M.E., Cruz D.H., West M.M., Hitchcock A.P., Pezolet M. (2007). *Nephila clavipes* spider dragline silk microstructure studied by scanning transmission X-ray microscopy. J. Am. Chem. Soc..

[15] Jiang P., Guo C., Lv T.Y., Xiao Y.H., Liao X.J., Thou B. (2011). Structure, composition and mechanical properties of the silk fibres of the egg case of the Joro spider, *Nephila clavata* (Araneae, Nephilidae). J. Biosci..

[16] Gosline J.M., Demont M.E., Denny M.W. (1986). The structure and properties of spider silk. Endeavour.

[17] Zax D.B., Armanios D.E., Horak S., Malowniak C., Yang Z.T. (2004). Variation of mechanical properties with amino acid content in the silk of *Nephila clavipes*. Biomacromolecules.

[18] Yu H., Yang J.L., Sun Y.X. (2015). Energy absorption of spider orb webs during prey capture: A mechanical analysis. J. Bionic Eng..

[19] Gosline J.M., Guerette P.A., Ortlepp C.S., Savage K.N. (1999). The mechanical design of spider silks: From fibroin sequence to mechanical function. J. Exp. Biol..

